# SGLT2 inhibitors in patients with HFpEF: how old is too old?

**DOI:** 10.20517/jca.2022.30

**Published:** 2022-07-20

**Authors:** Dan Tong

**Affiliations:** Department of Internal Medicine, Division of Cardiology, University of Texas Southwestern Medical Center, Dallas, TX 75390, USA

## Abstract

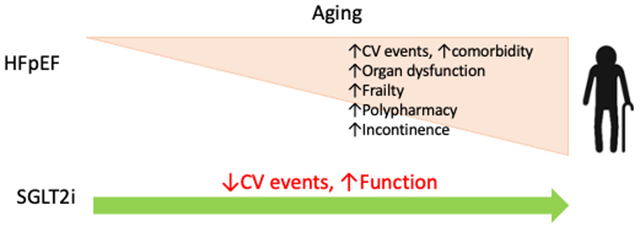

Heart failure with preserved ejection fraction (HFpEF) is increasingly recognized as a heterogeneous, systemic clinical syndrome. The complex nature of HFpEF makes it a diagnostic and therapeutic challenge. Recently, sodium-glucose cotransporter-2 (SGLT2) inhibitors have emerged as a promising therapy for HFpEF, as the EMPEROR-Preserved trial demonstrated that empagliflozin reduces the risk of the composite cardiovascular (CV) endpoint of death or heart failure (HF) hospitalization in HF patients with LVEF > 40%[1]. Therefore, SGLT2 inhibitors are listed in the 2022 AHA/ACC/HFSA HF guidelines as a class Ha recommendation for treating patients with HFpEF[2]. This marks a momentous and exciting advance in the management of HFpEF. However, many uncertainties remain while implementing this novel therapy into real-world clinical practice.

HFpEF is considered a geriatric syndrome, as most patients with HFpEF are ≥ 65 years old with multiple comorbidities and limited functional status[3]. Therefore, caring for HFpEF patients inevitably entails unique challenges. Given the increased burden of comorbidities and declining organ function associated with aging, will the benefits of SGLT2 inhibitors decline in the elderly? It is well known that older patients have poor tolerance to hypotension and hypoglycemia; will this make them particularly sensitive to side effects associated with SGLT2 inhibitors? Incontinence is common in the elderly and associated with an increased risk of urinary tract infection (UTI); will this pose a particular challenge for implementing SGLT2 inhibitors? Lastly, and perhaps most importantly, as HFpEF is associated with significant frailty and debilitation[4], it is critical to assess the impact of SGLT2 inhibitors on patient functional status. A recent study by Bohm *et al* provided valuable insights into these issues[5].

In a *post hoc* analysis of the EMPEROR-Preserved trial, these investigators examined the treatment effect size of empagliflozin across pre-specified age subgroups. Participants were divided into four groups (age < 65, 65-74, 74-80, and ≥ 80 years old). Eighty percent (4789 out of 5988) of participants were ≥ 65 years old, representing a typical HFpEF patient population. Among them, 27% (1299 out of 4789) were ≥ 80 years old, a group rarely represented in previous trials. As expected, patients of older age had a greater burden of comorbidities, including higher blood pressure, lower eGFR, and higher prevalence of atrial arrhythmias. Advanced age was associated with increased incidence of the primary composite outcome of CV death or HF hospitalization. The investigators discovered that the relative risk reduction with empagliflozin was similar across the age groups (HR 0.83 for < 65, 0.86 for 65-74, 0.72 for 75-79, and 0.73 for ≥ 80; *P* = 0.33), suggesting that the beneficial effect of empagliflozin is maintained across all age groups. Of note, prior analysis has suggested that empagliflozin has attenuated beneficial effects for patients with LVEF > 65%[6], and there are concerns that this group will be preferentially represented in the very elderly. The current study suggests that this is not correlated with age.

Preservation of function and independence is one of the core values of geriatric care and an important goal for HFpEF management[3]. Empagliflozin has been shown to improve health-related quality of life (HRQoL) measured by Kansas City Cardiomyopathy Questionnaire (KCCQ) scores in HFpEF patients regardless of baseline functional status[7]. In the current study, the authors demonstrated that this beneficial effect is again maintained across all ages[5]. Numerically, the improvement in KCCQ score appeared particularly early in the very elderly group (≥ 80), as compared with other age groups, and was maintained throughout the study period. This is of particular importance as poor functional status is strongly associated with mortality in the elderly[8]. Of note, although the overall average improvement in KCCQ score is modest (1-2 points), the authors pointed out that a significant proportion of patients achieved changes of > 5 points, which reflect clinically meaningful improvements in functional status.

Polypharmacy is common in older patients, and the elderly are particularly susceptible to medication-associated side effects. The majority of participants in the EMPEROR-Preserved trial were concurrently taking commonly prescribed CV medications, including ARB/ACEi, beta-blockers, and statins, representing a typical population seen in clinical practice[1]. As SGLT2 inhibitors increase glucose excretion in the urine, there are concerns regarding the risk of UTI and infections in the genital area in the elderly, given the high prevalence of incontinence in this population. Of note, no significant increases in the profile and incidence of side effects were observed between treatment and placebo groups across all ages, suggesting a satisfactory safety profile. Furthermore, the beneficial effect of empagliflozin in mitigating eGFR decline was similarly maintained across all age groups.

In summary, the study by Bohm *et al.* provides evidence that empagliflozin is efficacious and safe in patients with HFpEF across all adult age groups, including the very elderly (≥ 80 years old)[5]. The benefits of SGLT2 inhibitors observed in patients with heart failure across a wide spectrum of LVEF, diabetes status, baseline functional status, and age group[5–7,9] suggest its wide and continuously expanding clinical indication and marks a significant achievement in cardiovascular medicine. Whereas the exact molecular mechanisms underlying the beneficial effects of SGLT2 inhibitors remain unclear, their wide-ranging benefits raise the possibility that they work via fundamental cellular pathways that benefit health and longevity. Consistent with this notion, recent studies have demonstrated that SGLT2 inhibitors are intimately connected with pathways such as cell energy, senescence, autophagy, *etc*.[10]. Additional in-depth mechanistic studies are needed to elucidate molecular mechanisms governing SGLT2 inhibition-dependent benefits to the cardiovascular system.

## Data Availability

Not applicable.
